# Supplementary material for: Facile and Rapid Synthesis of Porous Hydrated V2O5 Nanoflakes for High-Performance Zinc Ion Battery Applications
Source: Nanomaterials (Basel). 2022 Jul 14;12(14):2400. doi: 10.3390/nano12142400 (PMC9318212; doi:10.3390/nano12142400)
Supplement: Supplementary file 1 [file nanomaterials-12-02400-s001.zip › nanomaterials-1800504-supplementary.pdf]

Supplementary Materials

Facile and Rapid Synthesis of Porous Hydrated V_2O_5 Nanoflakes for High-Performance Zinc Ion Battery Applications

Kai Guo [1,2,*], Wenchong Cheng [1], Haoxiong Chen [1], Hanbin Li [1], Jinxue Chen [1], Haiyuan Liu [1], Yunliang Tu [1], Wenhao She [1], Zhengkai Huang [1], Yinpeng Wan [1], Lixia Zou [1], Zhuyao Li [1], Xing Zhong [1], Yongchuan Wu [3], Xianfu Wang [4,*] and Neng Yu [1,*]

[1] Jiangxi Province Engineering Research Center of New Energy Technology and Equipment, School of Chemistry, Biology and Materials Science, East China University of Technology, Nanchang 330013, China; wenchongcheng@ecut.edu.cn (W.C.); chenhx@ecut.edu.cn (H.C.); hanbinli@ecut.edu.cn (H.L.); chenjinxue@ecut.edu.cn (J.C.); haiyuanliu@ecut.edu.cn (H.L.); yunliangtu@ecut.edu.cn (Y.T.); liming9889@ecut.edu.cn (W.S.); huangzhk@ecut.edu.cn (Z.H.); 2020330464@ecut.edu.cn (Y.W.); lxzou@ecut.edu.cn (L.Z.); lizhuyao@ecut.edu.cn (Z.L.); zhongxing@ecut.edu.cn (X.Z.);

[2] State Key Laboratory of Materials Processing and Die & Mould Technology, Huazhong University of Science and Technology, Wuhan 430000, China

[3] Jiangxi Key Laboratory for Mass Spectrometry and Instrumentation, East China University of Technology, Nanchang 330013, China; wuyoungchan@163.com

[4] State Key Laboratory of Electronic Thin Film and Integrated Devices, University of Electronic Science and Technology of China, Chengdu 610054, China

* Correspondence: guokai@ecut.edu.cn (K.G.); xfwang87@uestc.edu.cn (X.W.); neng063126@ecut.edu.cn (N.Y.)

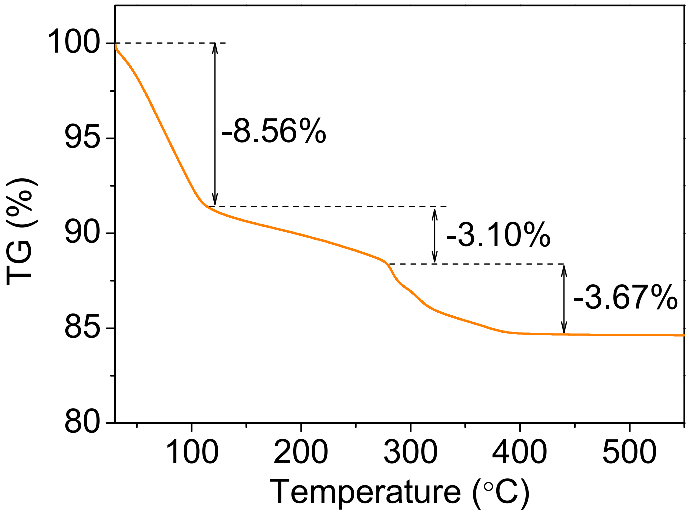

Figure S1. TG analysis of hydrated V_2O_5 nanomaterial.

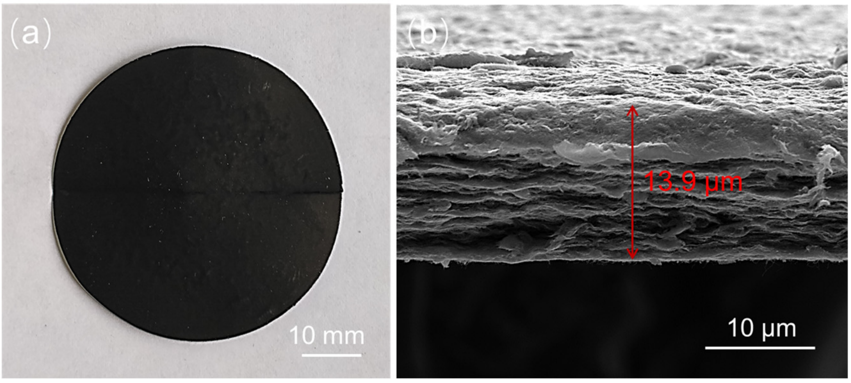

Figure S2. The structural characterization of $V_2O_5·nH_2O$/CNT composite film. (**a**) Photo and (**b**) cross-section SEM image of the composite film.

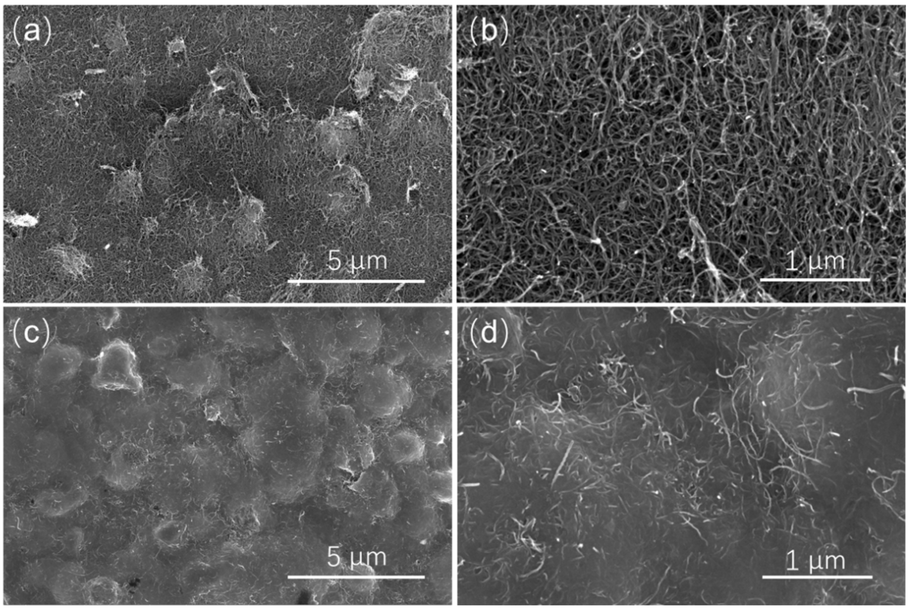

Figure S3. Morphology characterization of pure CNT film and $V_2O_5 \cdot nH_2O$/CNT composite film. (**a**) and (**b**) SEM images of pure CNT film. (**c**) and (**d**) SEM images of $V_2O_5 \cdot nH_2O$ /CNT composite film.

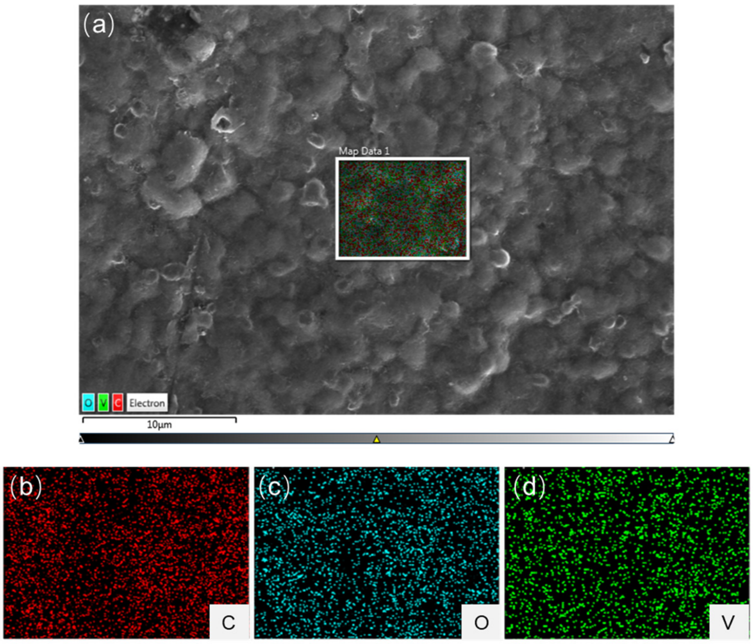

Figure S4. EDS characterization of $V_2O_5 \cdot nH_2O$/CNT composite film. (**a**) SEM image and, (**b**–**d**) carbon, oxygen, and vanadium element distribution in the selected area.
